# Carotid intima–media thickness and endothelial function in adolescents exposed to alcohol consumption and cigarette smoking *in utero*: a mediation analysis

**DOI:** 10.1186/s12872-025-05010-1

**Published:** 2025-08-07

**Authors:** Tammy Hartel, Aamer Sandoo, André Oelofse, Juléy De Smidt

**Affiliations:** 1 Department of Medical Biosciences, Faculty of Natural Sciences, University of the Western Cape, Private Bag X17, Bellville, Cape Town, 7530 South Africa; 2 School of Psychology and Sport Science, George Building, Bangor University, Bangor, LL57 2PZ UK

**Keywords:** Blood pressure, Body mass index mediators, Teratogens, Vascular dysfunction

## Abstract

**Background:**

Previous studies have shown the effects of teratogen exposure *in utero* on the vascular system, but its direct and indirect effects are still questionable. Therefore, this study explored the effects of potential mediators of teratogen exposure on carotid intima–media thickness (cIMT) and flow-mediated dilation (FMD).

**Methods:**

This study was conducted between January 2022 and August 2024 in Cape Town, South Africa, and included 307 adolescents (aged 10–14) and their mothers. Anthropometry data, lipid profiles, systolic (SBP) and diastolic blood pressure (DBP), physical activity and maternal health behaviours were obtained. Vascular measurements (left and right cIMT and brachial artery FMD) were obtained via ultrasound, which included analysing the peak artery diameter separately. Statistical analyses were performed via SPSS^®^ version 29, and mediation analyses were conducted via PROCESS Macros.

**Results:**

The cohort included a control group (*n* = 105), tobacco-exposed individuals (*n* = 115), dual-exposed individuals (tobacco and alcohol) (*n* = 73), and alcohol-exposed individuals (*n* = 14). A significant positive correlation was observed between alcohol exposure duration and LcIMT (rho = 0.531, *p* < 0.05), and a strong negative correlation was observed with peak diameter (rho=-0.788, *p* < 0.001), with a very strong correlation in males (*r*=-0.894, *p* < 0.05). The duration of alcohol exposure significantly affected LcIMT (*p* = 0.0468) after adjusting for body mass index (BMI), SBP and DBP. However, brachial artery (BA) peak diameter was not significantly associated with the duration of alcohol consumption during pregnancy after the adjustment for age, SBP or BA baseline diameter.

**Conclusion:**

Increased BMI and SBP may contribute to a reduced peak diameter in adolescents exposed to alcohol, potentially indicating lower FMD in adulthood. Further studies should elucidate the biological mechanisms involved through long-term prospective studies.

**Supplementary Information:**

The online version contains supplementary material available at 10.1186/s12872-025-05010-1.

## Introduction

In the Western Cape, South Africa, an alarming 20.8–56.3% of women use tobacco during pregnancy and 8.1% of women are among the mixed ethnic group [1]. In addition, 31.5–37.4% of prenatal smokers also consumed alcohol during pregnancy [1, 2]. In low socioeconomic communities, children aged 8–13 years old presenting with cardiovascular risk factors are at an increased risk of cardiovascular disease (CVD) and diabetes in adulthood [3]. Therefore, identifying the primary mediators of cardiovascular risk in children and adolescents, particularly those related to cigarette smoking and alcohol use during pregnancy, remains a crucial challenge for ongoing research [4]. During gestation, both alcohol and nicotine cross the placenta, potentially causing oxidative stress, decreasing nitric oxide (NO) synthesis, and leading to atherosclerosis [4, 5]. Therefore, vascular measurements such as cIMT and FMD allow non-invasive measurements of vascular structure and function and help identify subclinical atherosclerotic changes in the vasculature, which might be apparent in children and adolescents. Intima–media thickness is a measurement of both the intima and tunica media and is commonly assessed via B-mode ultrasound of the carotid arteries [6], whereas the FMD measures the endothelium-dependent response to reactive hyperemia via the release of the vasodilator molecule nitric oxide (NO) [7].

Most importantly, previous studies have shown that adolescents exposed to high levels of maternal smoking (≥ 5 cigarettes per day) have higher triglycerides, elevated SBP, and an increased risk of cardiometabolic clustering by the age of 10 [8–11]. Moreover, FMD was negatively correlated with BMI, waist circumference (WC), cIMT and visceral adipose tissue thickness in adults, suggesting that chronic inflammation caused by increased oxidative stress due to obesity as well as increased inflammatory cytokines play a significant role in vascular dysfunction as it may interfere with NO synthesis [12]. Other studies suggest deterioration of endothelial function by increased systemic inflammation and mitochondrial superoxide formation, mediated by increased tumor-necrosis factor alpha [13]. In adults, FMD was reportedly lower in alcohol users than in those who abstained from alcohol [14]. Increased markers detected in mothers with alcohol intake such as increased asymmetric dimethylarginine (ADMA) and homocysteine, may result in vascular and hemodynamic abnormalities through the inactivation of eNOS and nNOS [14]. Equally important, fetal alcohol exposure results in consistent changes in blood vessels, such as endothelial dysfunction and arterial stiffening, similar to those associated with nicotine exposure [4, 14–16]. Furthermore, previous studies reported significantly greater aortic intima–media thickness and vascular dysfunction in infants exposed to maternal smoking [17, 18] and greater cIMT in children exposed to both maternal smoking and alcohol use [16]. A prospective study using path analysis to study the effects of prenatal alcohol exposure (PAE) on adult vascular health revealed that PAE indirectly affects blood pressure through height and BMI. Similarly, PAE has a direct negative effect on endothelial function, as measured by the reactive hyperemia index (RHI) via peripheral arterial tonometry [19].

During gestation, both alcohol and nicotine cross the placenta, potentially causing oxidative stress, decreasing NO synthesis, and leading to atherosclerosis [4, 5]. In a South African study, asymmetric dimethylarginine (ADMA), a marker of endothelial function, was positively correlated with pulse wave velocity (PWV) and 8-hydroxy-2deoxyguanosine (8-OhdG) in children [20]. Asymmetric dimethylarginine inhibits nitric oxide synthase (eNOS), thereby preventing NO synthesis and reducing NO bioavailability [20]. Yzydorczyk et al. suggested that epigenetic changes can also control eNOS expression through DNA methylation, histone modification and microRNAs [21].

This observation was also reported in preclinical studies, where both moderate and binge drinking disrupted the eNOS pathway, resulting in vascular dysfunction in the uterine artery of rats [22, 23]. However, data from human studies on the effects of PAE on vascular outcomes in children and adolescents are lacking [24, 25]. Therefore, this study aimed to explore potential mediators in the development of subclinical atherosclerosis measured by cIMT and FMD in adolescents exposed to cigarette smoking and alcohol *in utero*. The study focused specifically on a low-income urban population as the prevalence of smoking and alcohol use during pregnancy is alarmingly high in these populations, particularly in South Africa [1, 2].

## Methods

### Study design and participant recruitment

The research design was a cross-sectional cohort study conducted between January 2022 and August 2024. A two-stage stratified random cluster sampling approach was used to randomly select four schools in a low socioeconomic population. A total of 307 participants were recruited, and targeted age groups (10–14 years) were assessed. Exclusions were due to having twins (4), missing interviews (7), or being a foster parent (1). Women and their children were interviewed face-to-face using a researcher-generated questionnaire to obtain their demographic characteristics, self-reported cigarette smoking and alcohol use during pregnancy, birth measurements, second-hand smoke (SHS) exposure, and family history of CVD. A Spearman’s correlation (rho = 0.896, *p* < 0.001) demonstrated a strong positive correlation between recalled birthweight and recorded birthweight, suggesting that women’s recall of birth weight was generally reliable. Maternal tobacco smoking included type of tobacco (cigarette, vape, cigars, water pipe (hookah), or chewed tobacco products), average number of cigarettes smoked per day, and smoking duration (gestational months and trimester). Alcohol use during pregnancy included drinking duration (gestational months and trimester), drinking frequency, type of alcohol, and number of drinks consumed in one sitting. The child’s physical activity and self-reported smoking and alcohol use were recorded. Serum cotinine was tested to verify self-reported smoking. Physical activity were classified on the basis of the FITT recommendations for children and adolescents by the American College of Sports Medicine (ACSM) guidelines for exercise testing and prescription (10th ed.) [26]. Physical activity was defined as participating in at least one hour of daily moderate–high-intensity physical activity. Socioeconomic status was determined on the basis of education level, occupation status, and household income. All questions and variables were guided by the South Africa 2022 census statistical release [27].

A pilot study was conducted on 30 healthy adolescents to ensure instrument and tester reliability and validity. All participants were tested at the same time of day to avoid diurnal variations. Three measurements were taken on all measurable study variables via standard and precision research equipment on two separate occasions. All instruments were calibrated regularly before use. Cronbach’s alpha reliability test showed that repeated measures of anthropometry and BP measurements indicated good to excellent reliability. Spearman’s rank correlation indicated good test‒retest reliability by the sonographer (rho = 0.834, *p* < 0.01).

### Anthropometric measurements

Body weight was measured in kilograms (kg) via a digital scale, and stature was measured in centimetres (cm) via a mechanical stadiometer. Body Mass Index was calculated by dividing weight (kg) by height (m^2^). Obesity was defined as a BMI-for-age of 2SD above the mean, and overweight was defined as a BMI-for-age of 1SD above the mean, according to sex and age [28–30]. Waist circumference was measured at the umbilicus level. Triceps skinfold thickness (tSFT) and subscapular skinfold thickness (sSFT) were measured using Holtain callipers according to the SANHANES clinical staff manual. The sum of the skinfold thickness (Sum SFT) was calculated by adding the tSFT and sSFT. All anthropometric measurements were measured three times, and the average was recorded as the final measurement.

### Clinical measurements

#### Blood pressure and lipogram test

Systolic and diastolic blood pressure were measured on the right upper arm after a five-minute rest period via an automated digital sphygmomanometer (Microlife, BP A200 AFIB) and pediatric-sized cuff. All measurements were taken three times, with three minutes between measurements. The mean values were recorded as the final measurement. Total cholesterol, LDL-cholesterol, HDL cholesterol, triglycerides and non-fasting blood glucose were measured via a handheld CardioChek *Plus* machine. The procedure was followed according to the user guide provided by CardioChek *Plus* (PTS diagnostics).

#### Ultrasound measurements

The cIMT ultrasound measurements were obtained from 103 participants and included 56 FMD measurements. The cIMT and FMD of the BA were performed by a blinded sonographer and analysed independently from the sonographer by a fully trained cardiovascular physiologist (AS). The cIMT was measured via B-mode ultrasonography (Voluson E8 ultrasound system, GE Healthcare, Kretz ultrasound, Zipf, Austria) and a 9 L-D (3.1–10 MHz) transducer. Measurements were obtained from the far wall of the common carotid artery. The left and right Common Carotid artery (CCA) intima-media thickness was measured using the Mannheim Consensus, an international protocol [31]. The right BA FMD was assessed via recent guidelines to evaluate endothelium-dependent vasodilation [32, 33]. Ultrasound measurements were taken in three periods: baseline measurements, vascular occlusion measurements, and reactive hyperemia measurements. The baseline period was recorded up to 60 s; for the cuff-occlusion period, the cuff pressure was placed on the forearm distal to the humeral epicondyle and was inflated to a suprasystal level (> 50 mmHg) above the resting SBP, and the wrist was occluded for three minutes. The post-occlusion period was recorded for at least three minutes using a frame grabber (Epiphan ESP1360). FMD was analysed via Medical Imaging Applications Brachial Analyser software, and the baseline diameter, peak diameter, and FMD percentage were obtained. The peak diameter was defined as the greatest change from the resting baseline diameter of the BA. According to the established guidelines, peak diameter is identified at any point within the 180 s after cuff deflation. This has been reported to be the most accurate approach in determining the peak diameter, especially as peak diameter can occur at different time points in different individuals [32].

### Covariates

Potential covariates were selected on the basis of previous literature [19, 34–36]. Maternal age, educational level, socioeconomic status, household smoking, maternal pre-pregnancy weight, cigarette smoking and alcohol use during pregnancy, sex, BMI, birthweight, childhood self-reported smoking status, serum cotinine levels and physical activity were obtained at enrolment from a researcher-generated questionnaire.

### Data analysis plan

Descriptive statistical analysis results are expressed as the means and standard deviations (95% CI). A Shapiro‒Wilk test was used to test for normality. Most of the data were not normally distributed; therefore, nonparametric tests were used (Spearman’s rank correlations, Kruskal‒Wallis H tests and Mann‒Whitney U tests). A *p* value < 0.05 and *p* value < 0.01 indicated statistical significance. ANCOVA was performed to determine the effects of maternal weight on adolescent birthweight and weight differences across the exposure groups.

Mediation analyses were performed via PROCESS Macros and linear regression to identify the main mediators [37]. An explanatory factor analysis was run to determine the contribution of each factor for the subsequent mediation analysis. Bartlett’s test of sphericity of *p* < 0.001 indicated the suitability of the data. Birthweight and BMI contributed 30.13% and 46.81% of the variance, respectively. Weight, SBP, WC and alcohol duration were also identified as contributing factors. We examined the relationship of peak diameter with teratogen exposure separately from FMD%, as exposure to alcohol and nicotine may have a depressive effect on peak diameter. For bootstrapped confidence intervals (CIs), 5000 bootstrap samples were selected with a 95% CI. The data are presented as tables and diagrams showing the relationships among the variables.

## Results

Compared with smoking mothers (U = 59.31 ± 17.62 kg, *p* < 0.035) and non-smoking mothers (U = 68.19 ± 17.53 kg), mothers who smoked cigarettes and consumed alcohol during pregnancy had significantly lower estimated pre-pregnancy weights (U = 52.18 ± 22.76 kg, *p* < 0.000) (Table 1). Birth weight was significantly different across all exposure groups (*p* < 0.05), which disappeared after adjusting for maternal pre-pregnancy weight (F (4, 128) = 0.049, *p* = 0.986, partial η^2^ = 0.001). Newborns in the dual-exposed group were, on average, 379.89 g lighter than nonexposed newborns, however, this was not significant (*p* = 0.053).

**Table 1:** Maternal and birth characteristics of the study participants

Maternal characteristics	Overall	Control	Nicotine exposed	Dual exposed	Alcohol exposed	Kruskal‒Wallis Test
	*N* = 307	*N* = 105(34.20%)	*N* = 115(37.46%)	*N* = 73(23.78%)	*N* = 14(4.56%)	*P* value
	*n* (%)	*n* (%)	*n* (%)	*n* (%)	*n* (%)	
Maternal age at birth, years (x̄ ± SD)	27.21 ± 8.39	27.65 ± 7.71	25.96 ± 8.19	27.86 ± 7.45	31.62 ± 15.84	0.248
Maternal prepregnancy weight, kg (x̄ ± SD)	60.12 ± 19.66	68.19 ± 17.53	59.31 ± 17.62^*^	52.18 ± 22.76^**^	59.4 ± 10.78	< 0.001^**^
Mother’s education, %						
Primary school/No high school certificate, %	28 (9.9%)	3 (3.2%)	17 (15.9%)	5 (7.4%)	3 (23.1%)	-
High school certificate, %	234 (82.7%)	75 (79.8%)	87 (81.3%)	62 (91.2%)	9 (69.2%)	-
Tertiary education, %	19 (6.7%)	15 (16.0%)	3 (2.6%)	0 (0%)	1 (7.7%)	-
Socioeconomic status, %						
Employment status, %	95 (33.3%)	41(43.6%)	33(30.6%)	15(21.7%)	5(38.5%)	-
Number of household members, mean	8.37 ± 4.4	7.97 ± 4.4	8.11 ± 3.7	9.23 ± 5.4	8.69 ± 3.8	-
Household income < R3500 (SA Rands)	176(61.8%)	41(13.36%)	77(25.08%)	47(15.31%)	11(3.58%)	-
Birth characteristics						
Birthweight(g)	3069.46 ± 752.39	3266.92 ± 876.35	2968.76 ± 604.51	2887.03 ± 655.01	3407.78 ± 965.08	0.023^*^

Note: *SD* standard deviation, *SA* Rands South African Rands

^*^Significant at the 0.05 level (2-tailed)

^**^Significant at the 0.01 level (2-tailed)

Table 2 shows the cardiometabolic and vascular measurements taken in the adolescents. Compared with the control group, the nicotine-exposed group had significantly lower weights (U = 38.77 ± 10.31 kg, *p* < 0.05). After controlling for maternal pre-pregnancy weight, there were no significant differences in adolescent weight (F (3,148) = 0.884, *p* = 0.451, partial η^2^ = 0.018). Adolescents in the nicotine (U = 145.71 ± 10.03 cm) and alcohol-exposed groups (U = 145.51 ± 11.99 cm) were on average shorter, followed by those in the dual-exposed group (U = 146.21 ± 8.61 cm, *p* < 0.05). The results indicated that SBP was significantly greater in the nicotine-exposed (U = 117.92 ± 14.29 mmHg, *p* < 0.01) and dual-exposed groups (U = 115.25 ± 14.99 mmHg, *p* < 0.05).

**Table 2:** Cardiometabolic and vascular measurements in adolescents according to exposure group

Characteristics	Overall	Control	Nicotine-exposed	Dual-exposed	Alcohol-exposed	Kruskal‒Wallis Test
	*N* = 307	*N* = 105 (34.2%)	*N* = 115 (37.4%)	*N* = 73 (23.8%)	*N* = 14 (4.6%)	*P* value
	*n* (%)	*n* (%)	*n* (%)	*n* (%)	*n* (%)	
Age (years) (x̄ ± SD)	11.6 ± 1.32	11.5 ± 1.23	11.59 ± 1.46	11.77 ± 1.1	11.71 ± 1.77	-
Male (%)	125(40.7)	41(39.1)	52(45.2)	29(39.7)	3(21.4)	-
BMI category, %						
Overweight (z score ≥ −1 & < 2)	35 (5.5%)	19(18.3%)	7(6.1%)	7(9.6%)	1(7.1%)	-
Obese (z score > 2)	28 (4.4%)	14(13.3%)	9(7.8%)	5(6.8%)	0(0%)	-
Cardiometabolic measurements, mean ± SD						
Weight (kg) (x̄ ± SD)	40.62 ± 11.67	43.19 ± 12.29	38.77 ± 10.31	40.19 ± 12.76	38.84 ± 9.27	0.029*
Height (cm) (x̄ ± SD)	146.52 ± 9.42	147.80 ± 9.42	145.71 ± 10.03	146.21 ± 8.61	145.51 ± 11.99	0.490
BMI (kg·m^2^) (x̄ ± SD)	18.70 ± 4.19	19.57 ± 4.54	18.09 ± 3.72	18.54 ± 4.54	18.06 ± 1.91	0.062
^*^Sum of skinfolds (mm) (x̄ ± SD)	19.66 ± 9.62	21.87 ± 10.84	18.51 ± 8.07	18.69 ± 10.15	17.59 ± 6.36	0.110
Waist circumference (cm) (x̄ ± SD)	63.65 ± 9.21	65.35 ± 9.64	62.68 ± 8.24	63.37 ± 10.38	60.63 ± 5.13	0.097
SBP (mmHg) (x̄ ± SD)	114.59 ± 13.92	110 ± 11.98	117.92 ± 14.29^**^	115.25 ± 14.99^*^	112.23 ± 11.87	0.001^**^
DBP (mmHg) (x̄ ± SD)	68.99 ± 10.59	67.64 ± 8.62	71.16 ± 12.71	67.74 ± 9.22	66.39 ± 8.11	0.051
Triglycerides (mmol/L) (x̄ ± SD)	1.41 ± 1.23	1.65 ± 1.35	1.22 ± 0.94	1.42 ± 1.02	1.13 ± 0.72	0.126
HDL cholesterol (mmol/L) (x̄ ± SD)	1.92 ± 0.89	2.10 ± 0.92	1.67 ± 0.79^*^	2.03 ± 0.91	2.05 ± 0.86	0.004^*^
LDL cholesterol (mmol/) (x̄ ± SD)	2.0 ± 1.28	2.0 ± 1.28	1.96 ± 1.33	2.04 ± 1.32	1.79 ± 0.67	0.462
Non-fasting blood glucose (mmol/L)	5.34 ± 0.86	5.34 ± 0.84	5.35 ± 0.85	5.36 ± 0.89	5.09 ± 0.98	0.641
Physical activity						
Physically active, %	48 (16.4%)	15(14.7%)	22(20.8%)	11(15.5%)	0(0%)	-
Low intensity, min·week, (x̄ ± SD)	100.64 ± 238.64	84.92 ± 205.51	101.41 ± 237.63	121.28 ± 275.65	108.08 ± 277.59	0.957
Moderate intensity, min·week (x̄ ± SD)	155.57 ± 329.14	146.39 ± 259.18	176.17 ± 416.50	144.54 ± 280.39	133.85 ± 299.15	0.848
High intensity, min·week (x̄ ± SD)	63.94 ± 209.86	73.45 ± 290.11	39.98 ± 112.20	90.33 ± 203.17^*^	34.62 ± 86.47	0.041^*^
Mod-High intensity, min·week (x̄ ± SD)	353.91 ± 656.84	339.20 ± 557.38	367.0 ± 805.37	367.53 ± 556.41	302.31 ± 598.31	0.548
Self-reported smoking, %	26(9.6%)	5(5.4%)	8(8.2%)	8(11.9%)	5(38.5%)	-
Cotinine levels (ug/L) (x̄ ± SD)	88.01 ± 78.17	120.77 ± 93.17	65.76 ± 68.96	84.88 ± 63.69	250 ± 0.00^#^	0.172
Self-reported alcohol consumption, %	7(2.6%)	1(4.3%)	1(1.0%)	2(3.0%)	0(0%)	-
Left cIMT(mm)(*n* = 103) (x̄ ± SD)	0.54 ± 0.08	0.52 ± 0.08	0.55 ± 0.08	0.54 ± 0.08	0.50 ± 0.00^#^	0.529
Right cIMT(mm)(*n* = 103) (x̄ ± SD)	0.55 ± 0.09	0.55 ± 0.09	0.55 ± 0.08	0.53 ± 0.07	0.56 ± 0.00^#^	0.454
Baseline diameter (mm) (*n* = 56) (x̄ ± SD)	2.97 ± 0.36	3.02 ± 0.399	2.94 ± 2.89	2.95 ± 0.42	2.90 ± 0.00^#^	0.490
Peak diameter (mm) (*n* = 56) (x̄ ± SD)	3.26 ± 0.37	3.29 ± 0.39	3.24 ± 0.35	3.29 ± 0.40	3.19 ± 0.00^#^	0.455
FMD% (*n* = 56) (x̄ ± SD)	10.51 ± 10.24	9.34 ± 10.53	10.61 ± 9.01	12.25 ± 12.53	10.00 ± 0.00^#^	0.458

Note: *SD* standard deviation, *BMI* Body mass index, *SBP* Systolic blood pressure, *DBP* Diastolic blood pressure, *LDL* Low-density cholesterol, *HDL* High-density cholesterol, *cIMT* Carotid intima media thickness, *FMD* Flow-mediated dilation %. ^*^Sum of skinfolds calculated as: average subscapular skinfold (mm) + average triceps skinfold (mm)

^*^Significant at the 0.05 level (2-tailed)

^**^Significant at the 0.01 level (2-tailed)

^#^Only one value – no standard deviation

Supplementary Table 1 also shows a positive correlation between BMI (rho = 0.451, *p* < 0.05), SBP (rho = 0.390, *p* < 0.05) and WC (rho = 0.443, *p* < 0.05) with BA peak diameter in females. As shown in Supplementary Table 2, females weighed significantly more than males did (U = 41.79 ± 11.68 kg, *p* < 0.01). However, males were significantly shorter than females were (U = 145.3 ± 9.76 cm, *p* < 0.05). In addition, BMI (U = 19.06 ± 4.35 kg·m^2^, *p* < 0.05) and SSFTs (U = 20.69 ± 9.61 mm, *p* < 0.01) were significantly greater in females than in males. As shown in Table 3, a significant and moderate positive correlation was observed between alcohol duration and LcIMT (rho = 0.531, *p* < 0.05), and a strong negative correlation was observed with peak diameter (rho = −0.788, *p* < 0.001), and this correlation was stronger in males, when separating sex (Table 3). However, with cIMT, no significant correlations were observed between LcIMT or RcIMT and the daily frequency or duration of cigarette smoking or alcohol consumption.

**Table 3:** Spearman’s rank correlations between teratogen exposure on cIMT and FMD values

	RcIMT	LcIMT	BA Baseline_	BA Peak_diameter	BA FMD%	Cig_Freq_day	Smoke_Dur	Alc_dur
LcIMT	.234^*^	-						
BA Baseline diameter	.003	-.017	-					
BA Peak_diameter	.024	-.146	.734^**^	-				
BA FMD%	.036	-.127	-.415^**^	.212	-			
Cig_Freq_day	-.072	-.072	.085	.009	-.106	-		
Smoke_dur	-.012	-.111	.068	.110	-.03	.280^**^	-	
Alc_dur	-.008	.531^*^	-.409	-.788^**^	-.151	-.042	.303^*^	-
Alcohol_Freq	-.021	-.115	-.255	-.185	-.104	.312^*^	-.096	-.026
Males								
LcIMT	0,12	-						
BA Baseline diameter	0,112	0,151	-					
BA Peak_diameter	0,202	0,053	,728^**^	-				
BA FMD%	-0,027	-0,068	-0,217	0,402	-			
Cig_Freq_day	-0,118	-0,382	0,139	-0,08	-0,379	-		
Smoke_dur	0	-0,118	-0,304	0,14	0,351	,268^*^	-	
Alc_dur	-0,356	,809^*^	-0,783	-,894^*^	0,112	-0,337	-0,026	-
Alcohol_Freq	-0,009	-0,087	-0,038	0,053	0,143	,262^*^	-0,005	-0,239
Females								
LcIMT	,305^*^	-						
BA Baseline diameter	-0,093	-0,204	-					
BA Peak_diameter	-0,085	-0,325	,740^**^	-				
BA FMD%	0,083	-0,09	-,641^**^	-0,035	-			
Cig_Freq_day	-0,162	0,128	-0,025	-0,146	0,22	-		
Smoke_dur	-0,107	-0,163	0,009	-0,072	-0,151	0,072	-	
Alc_dur	0,208	0,308	0,148	-0,577	-0,289	0,105	,515^**^	-
Alcohol_Freq	0,018	0,02	-0,08	-0,001	0,064	,313^**^	-0,124	0,177

*RcIMT* Right cIMT, LcIMT Left cIMT, *BA* Brachial artery, *FMD%* Flow-mediated dilation %, *Cig_day_day* cigarette frequency per day, *Smoke_dur* Smoking duration (months), *Alc_dur* Alcohol duration (trimesters), *Alcohol Freq* Alcohol frequency-Beer/wine_number_week

^*^Correlation is significant at the 0.05 level (2-tailed)

^**^Correlation is significant at the 0.01 level (2-tailed)

**Fig. 1:**
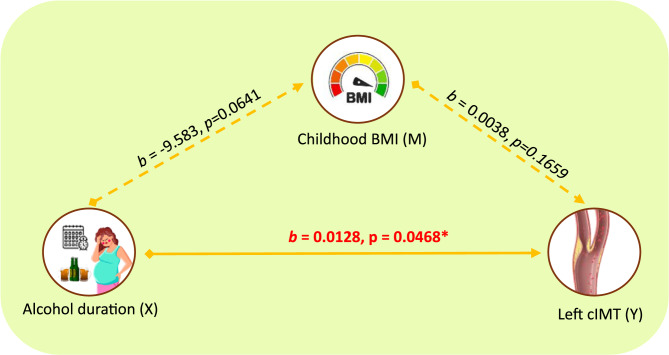
Mediation Model with Unstandardized Coefficients(*b*): Duration of alcohol consumption (trimesters) → childhood BMI → Left cIMT. X: Independent variable, Y: Dependent/Outcome variable, M: Potential mediator. Solid lines indicate direct path. Dashed lines indicate direct paths that involve a mediator

Figure 1 illustrates the direct and indirect effects of alcohol duration(trimesters) on LcIMT. The mediation pathway for RcIMT and teratogen exposure was not further investigated, as no significant correlations or direct effects were found. Similar to LcIMT, the direct effect of the duration of maternal alcohol consumption on LcIMT was significant (*b* = 0.0128, SE = 0.0060, t = 2.1437, *p* < 0.05), with a 95% CI of 0.0002, 0.0255, and the significance was not attenuated after adjusting for the child’s BMI, SBP and DBP.

**Fig. 2:**
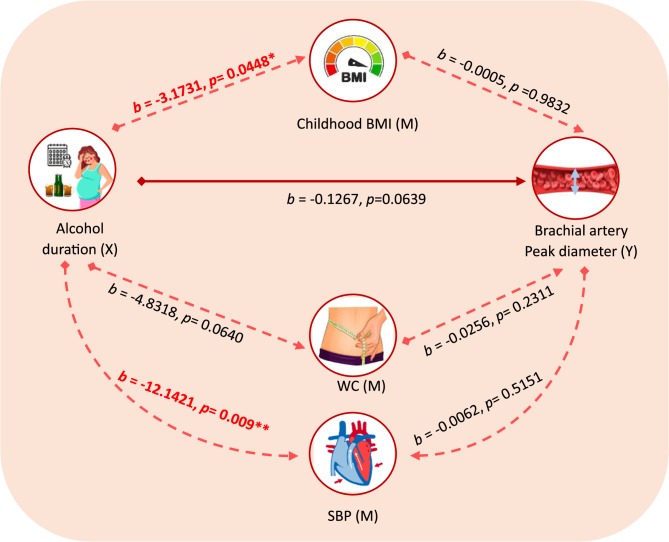
Parallel Mediation Model with Unstandardized Coefficients(*b*): Alcohol duration(trimesters) → Childhood BMI/WC → Brachial artery peak diameter. X: Independent variable, Y: Dependent/Outcome variable, M: Potential mediator. Solid lines indicate direct path. Dashed lines indicate direct paths that involve a mediator

Figure 2 illustrates the direct and indirect effects of alcohol duration on the BA peak diameter. The brachial artery peak diameter after cuff release was significantly associated with the duration of alcohol consumption during pregnancy (trimesters) (*b* = −0.2820, SE = 0.0454, t = −1.2220 *p* < 0.01). However, the significance was lost after adjustment for age, SBP and BA baseline diameter. Similarly, alcohol duration (trimesters) did not significantly affect the BA peak diameter (*b* = −0.2042, SE = 0.1065, t = −1.9176, *p* = 0.1133). Similar to BMI, a significant association was observed with peak BA diameter (*b* = −0.0368, SE = 0.0417, t = −2.4946, *p* < 0.05), but this association was lost after adjusting for SBP and baseline diameter.

## Discussion

The aim of this study was to explore potential mediators in the development of subclinical atherosclerosis measured by cIMT and FMD in adolescents exposed to cigarette smoking and alcohol *in utero* in a low-income urban population. We have identified increased BMI and SBP as potential mediators in reduced peak diameter in adolescents exposed to alcohol. However, BMI was not a mediator between alcohol duration and LcIMT. The direct effects of maternal alcohol and smoking on the vascular outcomes will also be discussed.

In the current study, cIMT was not significantly different across exposure groups, similar to previous studies [38, 39]. Whereas, in a previous study, a significantly greater cIMT was reported in five-year-old children exposed to maternal smoking (18.8 μm, *p* = 0.04) [16]. Furthermore, LcIMT was significantly correlated with alcohol duration in our study. This result may indicate that for each additional trimester or increase in the duration of maternal alcohol consumption, adolescent LcIMT increases by approximately 0.0128 mm. The left carotid artery may be more vulnerable to vascular wall changes as the artery originates directly from the aortic arch (therefore greater hemodynamic stress). Vascular damage may also be asymmetrical which may explain why there was a correlation with only the left carotid artery. In the Young Finns study, the frequency of wine and strong alcoholic beverages was also significantly correlated with cIMT after adjustment for sex, age and conventional risk factors [34], supporting the correlation found in this study. According to a systematic review, increased cIMT in children have also been associated with elevated SBP and obesity [36]. However, these confounding factors, specifically BMI, SBP and DBP were adjusted for in our study. Furthermore, a long prospective cohort study revealed that SBP at different life stages, infancy (weighting of 25.3%), preschool childhood (weighing of 27.0%), childhood (weighing of 18.0%), adolescence (weighing of 13.5%) and young adulthood (weighing of 16.2%), was associated with cIMT in adulthood [40]. Therefore, elevated SBP in adolescents may also affect cIMT in adulthood.

Furthermore, the results of the current study indicated that there was a negative correlation between alcohol duration and adolescent SBP, suggesting a protective effect of alcohol. In another study, 3–17-year-olds with prenatal alcohol exposure had a decreased risk for systolic and diastolic prehypertension; no association was reported between alcohol exposure and childhood BMI [41]. Arnold et al. [41], who were the first authors to report this unexpected outcome, suggested a possible explanation for this, through which alcohol creates an inflammatory response, triggering an increase in NO synthase expression, resulting in vasodilation and therefore decreased blood pressure. Similarly, in a more recent prospective cohort study, prenatal alcohol exposure had a protective effect on blood pressure, which may be explained through its ability to lower body weight and height [19]. However, these results should be interpreted with caution in terms of the general population [19]. Furthermore, we found that SBP as well as FMD and BMI were not independently associated with peak BA diameter. However, a study conducted on 52 adolescents aged 9–17 years reported a significantly lower FMD% in hypertensive adolescents (8.68%) than in normotensive adolescents (10.22%), indicating that even in childhood, blood pressure may reduce endothelium-dependent vasodilation, independent of age, BMI, lipid profile and sex [35]. Moreover, endothelial dysfunction was associated with obesity, high blood pressure and oxidative stress in 6–9-year-old South African children [20].

In this study, adolescents with teratogen exposure had healthy vascular function, as indicated by FMD values above 10% [42]. Although endothelial dysfunction measured by a reduced FMD% or significant differences were not observed in this cohort, this topic is worth further investigation, as the sample size for the ultrasound measurements was small and may have influenced the data. Furthermore, in a previous study, reduced endothelial function was significantly associated with low birth weight (coefficient = 50.18 kg, 95% CI 0.004 to 0.35, *P* = 0.04) [43]. For example, a 1 kg decrease in birthweight led to a reduced FMD value that was equivalent to an artery of an individual smoking 4.5 cigarette pack-years [43]. Moreover, children born at very low birth weights and preterm had significantly lower FMD% than did children with normal birth weights [44]. On the contrary, this association was not observed in our study, which may be attributed to the smaller sample size. However, there was a very strong correlation observed between alcohol duration and peak diameter in adolescent males. Previous studies have reported an association between prenatal alcohol exposure and structural anomalies, including cardiac malformations and vascular endothelial dysfunction, in the foetus [15, 45, 46]. Proposed mechanisms of prenatal alcohol on fetal vasculature include reduced umbilical cord and fetal cerebral blood flow, altered expression of genes associated with angiogenesis and fetal vascular development, decreased oxygen perfusion in fetal vasculature [19, 47–49]. Ethanol promotes vascular dysfunction through the generation of vascular reactive oxygen species (ROS), thereby increasing the level of oxidative stress, the main mediator [50]. Padovan et al. [50] also reported that ROS trigger the accumulation of intracellular Ca^2+^ ions (promoting hypercontractility), reduce NO bioavailability, and activate mitogen-activated protein kinases, leading to vascular injury and endothelial dysfunction. Similarly, nicotine also compromises NO bioavailability in the foetus [18]. In a prospective cohort study, path analysis suggested that prenatal alcohol exposure had a direct negative effect on vascular function in adulthood and an indirect effect on BP through BMI and height [19]. Interestingly, adults exposed to alcohol *in utero* weighed less and were shorter than those with no exposure [19]. These results were similar to what we found in the present study, where adolescents exposed to alcohol, and in certain instances, nicotine, were shorter than those who were not exposed, similar to a previous study [51]. The mothers were also smaller in terms of weight compared to mothers with higher weights in the control group. A possible explanation for this is that mothers who smoke and consume alcohol during pregnancy are generally smaller which may be explained by their lower socioeconomic status such as lower household income, lower education levels and higher unemployment rates, as well as smoking and alcohol consumption in previous generations, resulting in low birthweight infants and therefore smaller adolescents as they develop in a low-socioeconomic environment [51, 52]. Therefore, small mothers give birth to small infants [51, 52].

According to a few studies that established reference data for children and adolescents, normal FMD% in children fall between 7.7 and 8.2% [7], between 8.29 and 8.80% between 8–13-year-old females, and between 8.34 and 8.77% among 8–14-year-old males [53]. In children and adolescents, on average, the BA baseline diameter is generally smaller in females (2.96 mm) than in males (3.19 mm) and increases with age in both sexes [7, 53, 54]. Therefore, the size of the BA may be mediated by growth, such as weight and height [7]. However, Li et al. [53] suggested that there is age- and sex-specific endothelial function, as females had higher FMD% at 12–13 years of age, which may be attributed to higher estrogen levels during puberty, which are associated with better vascular function [53]. In this study, when sex was compared, there were no significant differences in FMD% between males and females, although females had a smaller FMD%, similar to previous studies reporting FMD rates of 11.9% and 12.7% in 6–16-year-old girls and boys, respectively [55]. However, the females in this study had a much smaller FMD (8.53%), whereas the males had similar measurements (11.26%) compared with previous studies [55].

Equally important, estrogen and testosterone increase during puberty, which may improve vascular function during that period, as FMD% is highest in females during puberty and slowly declines from 14 years of age [7, 53]. Furthermore, age at menarche is also related to baseline diameter; and therefore, changes in estrogen may play a role in regulating the arterial diameter [7]. This may explain why females had somewhat smaller baseline diameters in our study; however, according to Hopkins et al. [7], endothelial function does not differ by Tanner stage [7]. In this study, males also had higher baseline and peak diameters than females did [55], as expected. In fact, previous studies reported significant correlations between FMD and BMI, WC, age, systolic and diastolic BP, Tanner stage and carotid extra-media thickness (cEMT) [53]. The strengths of this study include the use of a fairly large sample for a comparative study. The study design minimized the influence of external factors by using inclusion and exclusion criteria and adjusting for potential confounders. Limitations of this study included the possibility of recall bias: under- or overestimations with recall of maternal smoking and drinking habits. However, the recall was previously validated with meconium metabolite analysis to confirm the concentrations [51]. We could not control for maternal preeclampsia and gestational hypertension in this cohort as majority of the mothers in the community did not report having preeclampsia during their pregnancy and only ten reported gestational hypertension. Physical activity of the mothers was also not recorded as it was beyond the scope of this study. Another limitation included a smaller-than-expected sample size for the FMD measurements, as image quality was affected by movement during the post-cuff period due to the young age of the cohort. In addition, several participants were lost to follow-up; therefore, the ultrasound videos could not be redone.

## Conclusion

The study findings suggest that an increase in BMI and SBP may be contributing factors to a reduced BA peak diameter in adolescents exposed to alcohol during pregnancy. The duration of alcohol exposure also significantly affected LcIMT, which may increase their CVD risk over their lifetime. Fortunately, children exposed to cigarette smoking and alcohol use *in utero*, whether born small or large, can prevent the development of CVD risk by managing BMI and BP. Larger, prospective studies are needed in low socioeconomic populations to further investigate the effects of nicotine and alcohol on vascular structure and function in adolescents. Therefore, providing evidence-based education to women about the harmful effects of alcohol and cigarette smoking is crucial to promote optimal cardiometabolic health in their children.

## Supplementary Information

Supplementary Material 1.

Supplementary Material 2.

## Data Availability

The data that support the findings of this study are available from the corresponding author upon reasonable request.
