# Supplementary material for: Carotid intima–media thickness and endothelial function in adolescents exposed to alcohol consumption and cigarette smoking in utero: a mediation analysis
Source: BMC Cardiovasc Disord. 2025 Aug 7;25:590. doi: 10.1186/s12872-025-05010-1 (PMC12330117; doi:10.1186/s12872-025-05010-1)
Supplement: Supplementary file 1 — Supplementary Material 1. [file 12872_2025_5010_MOESM1_ESM.docx]

**Carotid intima-media thickness and endothelial function in adolescents exposed to alcohol consumption and cigarette smoking *in utero*: a mediation analysis**

Tammy C Hartel^*1^, Aamer Sandoo^2^, André Oelofse^1^, Juléy JA De Smidt^1^

^1^Department of Medical Biosciences, Faculty of Natural Sciences, University of the Western Cape, Private Bag X17, Bellville, 7530, South Africa.

^2^School of Psychology and Sport Science, George Building, Bangor University, Bangor, LL57 2PZ, United Kingdom.

*Corresponding Author:

Tammy Hartel, Department of Medical Bioscience, Faculty of Natural Sciences, University of the Western Cape, Private Bag X17, Bellville, 7530, Cape Town, South Africa.

Tel: 27+ 82 778 1491

Email: [3366278@myuwc.ac.za](mailto:3366278@myuwc.ac.za)

ORCID iD: Tammy Hartel <https://orcid.org/0000-0003-2154-5379>

**Supplementary table 1**

| **Supplementary table 1: Spearman’s rank correlations between vascular and cardiometabolic measures across sex.** | | | | | | |
| --- | --- | --- | --- | --- | --- | --- |
|  |  | RcIMT | LcIMT | BA Baseline_  diameter | BA Peak_ diameter | BA FMD% |
| BMI | Males | -0,77 | 0.190 | 0.274 | 0.218 | 0.003 |
|  | Females | -0,214 | -0.220 | 0.435* | 0.451* | -0.230 |
| Total min Mod-High intensity | Males | -0,082 | 0.098 | 0.090 | 0.160 | 0.088 |
|  | Females | 0,059 | -0.035 | -0.013 | -0.061 | -0.055 |
| Average WC | Males | 0,035 | 0.184 | 0.301 | 0.182 | -0.103 |
|  | Females | -1,99 | -0.232 | 0.443* | 0.415* | -0.236 |
| Sum_SFT | Males | 0,053 | 0.101 | 0.251 | 0.317 | 0.133 |
|  | Females | -0,123 | -0.148 | 0.232 | 0.082 | -0.056 |
| Average SBP | Males | -,0,39 | 0.330* | -0.149 | -0.043 | 0.236 |
|  | Females | -0,257 | -0.155 | 0.167 | 0.390* | 0.209 |
| Average DBP | Males | -,210 | 0.179 | -0.291 | -.098 | 0.301 |
|  | Females | -0,288* | -0.093 | 0.134 | 0.183 | -0.009 |
| Triglycerides | Males | 0,150 | 0.027 | 0.197 | 0.058 | -0.063 |
|  | Females | -0,117 | 0.029 | 0.133 | 0.198 | -0.025 |
| HDL cholesterol | Males | -1,121 | -0.216 | 0.205 | -0.005 | -0.583 |
|  | Females | -0,189 | 0.003 | -0.112 | 0.050 | 0.336 |
| LDL cholesterol | Males | -0,167 | -0.022 | -0.027 | -0.159 | 0.971 |
|  | Females | -0,313* | -0.100 | 0.010 | 0.214 | 0.233 |
| Note: BMI Body mass index, Average WC Average waist circumference, SBP Systolic blood pressure, DBP Diastolic blood pressure, HDL High-density cholesterol, LDL Low-density cholesterol.  *Sum of skinfolds calculated as: average subscapular skinfold (mm) + average triceps skinfold (mm). | | | | | | |
