# Supplementary material for: Carotid intima–media thickness and endothelial function in adolescents exposed to alcohol consumption and cigarette smoking in utero: a mediation analysis
Source: BMC Cardiovasc Disord. 2025 Aug 7;25:590. doi: 10.1186/s12872-025-05010-1 (PMC12330117; doi:10.1186/s12872-025-05010-1)
Supplement: Supplementary file 2 — Supplementary Material 2. [file 12872_2025_5010_MOESM2_ESM.docx]

**Carotid intima-media thickness and endothelial function in adolescents exposed to alcohol consumption and cigarette smoking *in utero*: a mediation analysis**

Tammy C Hartel^*1^, Aamer Sandoo^2^, André Oelofse^1^, Juléy JA De Smidt^1^

^1^Department of Medical Biosciences, Faculty of Natural Sciences, University of the Western Cape, Private Bag X17, Bellville, 7530, South Africa.

^2^School of Psychology and Sport Science, George Building, Bangor University, Bangor, LL57 2PZ, United Kingdom.

*Corresponding Author:

Tammy Hartel, Department of Medical Bioscience, Faculty of Natural Sciences, University of the Western Cape, Private Bag X17, Bellville, 7530, Cape Town, South Africa.

Tel: 27+ 82 778 1491

Email: [3366278@myuwc.ac.za](mailto:3366278@myuwc.ac.za)

ORCID iD: Tammy Hartel <https://orcid.org/0000-0003-2154-5379>

**Supplementary table 2**

| **Supplementary table 2: Cardiovascular and vascular measurements across sex.** | | | |
| --- | --- | --- | --- |
|  | **Males** | **Females** | **Mann-Whitney-U-test** |
|  | **N= 125 (40.6%)** | **N= 183 (59.4%)** | **P value** |
| **Cardiometabolic measurements, mean ± SD** |  |  |  |
| Weight (Kg) (*x̄* ± SD) | 38.92 ± 11.49 | 41.79 ± 11.68 | 0.008** |
| Height (cm) (*x̄* ± SD) | 145.30 ± 9.76 | 147.36 ± 9.12 | 0.028* |
| BMI (Kg·m^2^) (*x̄* ± SD) | 18.18 ± 3.89 | 19.06 ± 4.35 | 0.032* |
| *Sum of skinfolds (mm) (*x̄* ± SD) | 18.16 ± 9.45 | 20.69 ± 9.61 | 0.001** |
| Waist circumference (cm) (*x̄* ± SD) | 63.59 ± 9.89 | 63.68 ± 8.75 | 0.522 |
| SBP (mmHg) (*x̄* ± SD) | 114.00 ± 14.84 | 114.98 ± 13.27 | 0.323 |
| DBP (mmHg) (*x̄* ± SD) | 68.58 ± 11.86 | 69.27 ± 9.67 | 0.222 |
| Triglycerides (mmol/L) (*x̄* ± SD) | 1.47 ± 1.19 | 1.37 ± 1.07 | 0.693 |
| HDL cholesterol (mmol/L) (*x̄* ± SD) | 1.92 ± 0.88 | 1.92 ± 0.90 | 0.713 |
| LDL cholesterol (mmol/) (*x̄* ± SD) | 1.97 ± 1.32 | 2.02 ± 1.24 | 0.277 |
| Non-fasting blood glucose (mmol/L) | 5.39 ± 0.79 | 5.29 ±0.90 | 0.068 |
| Cotinine levels (ug/L) | 101.13 ± 76.83 | 79.23 ± 81.17 | 0.384 |
| Left cIMT(mm) | 0.53 ± 0.07 | 0.54 ± 0.08 | 0.767 |
| Right cIMT(mm) | 0.56 ± 0.09 | 0.53 ± 0.08 | 0.112 |
| Baseline diameter (mm) (n=56) | 3.02 ± 0.39 | 2.95 ±0.34 | 0.599 |
| Peak diameter (mm) (n=56) | 3.35 ± 0.48 | 3.19 ± 0.29 | 0.133 |
| FMD% (n=56) | 11.26 ± 9.46 | 8.53 ± 8.96 | 0.363 |
| Note: SD standard deviation, BMI Body mass index, SBP Systolic blood pressure, DBP Diastolic blood pressure, LDL Low-density cholesterol, HDL High-density cholesterol, cIMT Carotid intima media thickness, FMD% Flow-mediated dilation %.  *Sum of skinfolds calculated as: average subscapular skinfold (mm) + average triceps skinfold (mm). | | | |
